# Comprehensive Genetic Analysis of Monokaryon and Dikaryon Populations Provides Insight Into Cross-Breeding of *Flammulina filiformis*

**DOI:** 10.3389/fmicb.2022.887259

**Published:** 2022-07-05

**Authors:** Hui Li, Lei Shi, Weiqi Tang, Weiwei Xia, Yingli Zhong, Xinyu Xu, Baogui Xie, Yongxin Tao

**Affiliations:** ^1^Institute of Cash Crops, Hebei Academy of Agriculture and Forestry Sciences, Shijiazhuang, China; ^2^College of Horticulture, Fujian Agriculture and Forestry University, Fuzhou, China; ^3^Mycological Research Center, Fujian Agriculture and Forestry University, Fuzhou, China; ^4^Marine and Agricultural Biotechnology Laboratory, Fuzhou Institute of Oceanography, Minjiang University, Fuzhou, China

**Keywords:** *Flammulina filiformis*, population structure, monokaryon, dikaryon, single nucleotide polymorphism, principal component analysis

## Abstract

*Flammulina filiformis*, as one of the most popular edible fungi in East Asia, is produced in an industrialized and standardized way. However, its monotonous variety and product convergence have seriously restricted the development of the industry. In this study, 11 cultivated strains and 13 wild strains of *F. filiformis* were collected from multiple regions of China and Japan and were performed genome sequencing. Together with genome data of six strains previously released, in total 23 dikaryons (formed by two monokaryons mating, can making fruiting body), 35 monokaryons (formed by protoplast-regenerating of dikaryon and isolating) were used for genetic diversity and population structure analysis based on the high-throughput genotyping. Firstly, a set of SNP markers with intrapopulation polymorphism including 849,987 bi-allelic SNPs were developed and basically covered all of 11 chromosomes with a high distribution density of 24.16 SNP markers per kb. The cultivated dikaryotic strains were divided into three subgroups, and their breeding history was made inferences, which is consistent with the available pedigree records. The wild dikaryotic strains were divided into two subgroups and showed varied contributions of genetic components with high genetic diversity. All the investigated dikaryons have a symmetric distribution pattern with their two constituent monokaryons in principal component analysis. Finally, we summarized the pedigree relationship diagram of *F. filiformis* main strains including six modules, and the genotypes of hybrids can be directly phased by the known parental allele according to it. This study provides a method to distinguish two sets of monokaryon haplotypes, and several valuable genetic resources of wild *F. filiformis*, and an effective strategy for guiding *F. filiformis* breeding based on the population structure and pedigree relationship in future.

## Introduction

*Flammulina filiformis* (Dai and Yang, 2018), known as winter mushroom or enokitake, belongs to the family Physalacriaceae, Agaricales (Ge et al., 2015). *F. filiformis* from eastern Asia differs from the European *F. velutipes* according to recent phylogenetic results based on multi-gene markers and morphological comparisons (Wang et al., 2018). *F. filiformis* is a widely cultivated and consumed edible fungus with high dietary and medicinal value (Rahman et al., 2015; Tang et al., 2016; Chen et al., 2020). It is also one of the most professional and productive edible fungi in industrialized cultivation (Bao et al., 2009). In nature, the fruiting body of wild *F. filiformis* has a hypertrophied pileus and a short hairy stipe. All wild *F. filiformis* are yellowish or brown in color (the degree of yellow varies by growth environment; Wang et al., 2018; Sharma et al., 2021). In 1928, the wild *F. filiformis* was first domesticated and artificially cultivated in Japan (Chen and Huang, 2013). The stipe was found to be more palatable during the breeding process, therefore *F. filiformis* with a longer stipe was cultivated (Sharma et al., 2021). *F. filiformis* without fluff on the stipe was also selectively cultivated because the hairy stipe of wild *F. filiformis* was found to have a bad taste (Tan et al., 2016). During cultivation, a white mutant sometimes appeared, and this led to production of the white *F. filiformis* variety. In the initial stage of artificial *F. filiformis* cultivation, the yellow *F. filiformis* was dominant, however, its production was gradually supplanted by the white strain. Almost all *F. filiformis* currently produced in industrialized cultivation is of the single white variety and there is a lack of diversification. Rising production costs and the increase in per unit area yield have created challenges that impede the development of the *F. filiformis* industry.

*Flammulina* *filiformis* breeding has been carried out extensively for many years, mainly through cross-breeding (Guo, 1997; Liu et al., 2018). Among existing strains, although the white variety generally has the characteristics of adhesion and aggregation at the base of the stipe, it also carries undesirable characteristics such as a highly fibrotic stipe that is not easy to chew. The higher yield of the white variety gives it an advantage in the market and during industrialized production. In contrast, both wild and cultivated strains of the yellow variety, generally have reduced adhesion and high dispersion at the base of the stipe, but they also have the desirable characteristic of a less fibrotic stipe that taste good and is easy to chew. The unconsolidated stipe has meant that the yellow variety has little advantage in yield over the white variety. As a result, many wild yellow strains were eliminated to optimize yield such that, the breeding parents now tend to be concentrated in a few white strains from prior *F. filiformis* breeding. The white variety is now widely used for industrialized cultivation and breeding has become monotonic and convergent.

Producing a variety of *F. filiformis* with diverse characteristics such as color, taste, and environmental adaptability will require an extensive collection and in-depth analysis, of potential *F. filiformis* parents with good traits that can be used for breeding. The whole population of *F. filiformis* and the genetic relationships between different varieties will need to be explored to construct a population structure and family relationship map for guiding the selection of parents (Liu et al., 2016; Bao, 2020). Some recent studies have assessed the population structure of *F. filiformis* strains (Lu et al., 2015; Shen et al., 2020; Gao et al., 2021). However, strains collected by previous studies are not representative enough of the *F. filiformis* population because they are composed of few wild germplasm resources. In addition, a comprehensive and systematic analysis of the genetic relationship between dikaryons and the constituent monokaryons has not yet been conducted. Simple sequence repeats (SSR) that were used as molecular markers in previous studies are not an ideal method for population structure analysis (Yang et al., 2019). Zhang et al. (2021) used genome-wide association studies (GWAS) to investigate the population of *Lentinula edodes* (Zhang et al., 2021), and markers based on genomic single nucleotide polymorphisms (SNP) were used to study *Agaricus bisporus* population structure (An et al., 2021). SNP markers showed similar grouping patterns and more neighborhood coordinate pedigree classifications for population structure analysis. At present, there is no genome-wide SNP marker for population structure analysis of *F. filiformis*.

This study expanded the collection of wild *F. filiformis* germplasm resources and sequenced the monokaryon and dikaryons genomes from many *F. filiformis* strains. The genetic diversity and population structure of monokaryons, dikaryons, and their combined populations were also analyzed. By establishing a *F. filiformis* population model, the genetic pedigrees and genotypes of the *F. filiformis* dikaryotic strains can be predicted, helping to guide effective cross-breeding.

## Materials and Methods

### Strains

**Figure 1:**
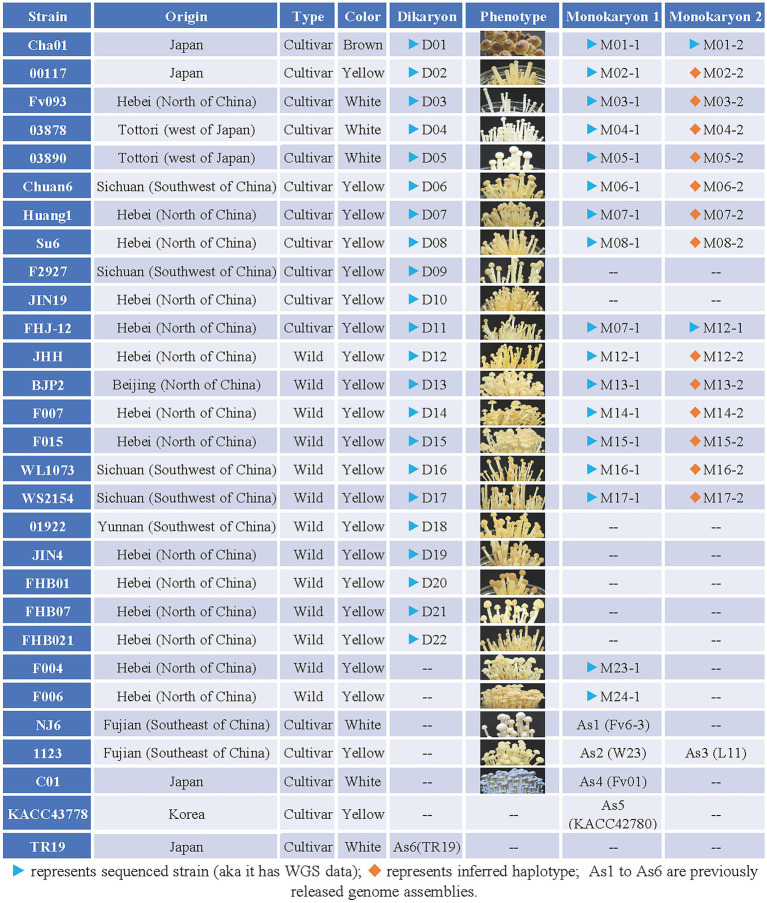
Collection of *Flammulina filiformis* strains information table. “Origin” represent the origin of the strain; “Type” represent the strain is wild type or cultivated type, wild type refers to strains collected in the field, while cultivated type refers to commercially available strains used for commercial production; “Color” represent the fruiting body of *F. filiformis* strain; “Dikaryon” and “Monokaryon” represent the Dikaryon strain and its associated two mononuclear strains; “Phenotype” represent the phenotype at the stage of the fruiting body of the strain.

*Flammulina filiformis* strain Cha01 (Serial number: D01, D represents dikaryon) and its two constituent monokaryotic strains CHA-Y-4 (M01-1, M represents monokaryon) and CHA-Y-25 (M01-2); 00117 (D02) and its constituent monokaryon 00117-Y-1 (M02-1); Fv093 (D03) and its constituent monokaryon Fv093-Y-B (M03-1); 03878 (D04) and its constituent monokaryon 03878-Y-1 (M04-1); 03890 (D05) and its constituent monokaryon 03890-Y-1 (M05-1); Chuan6 (D06) and its constituent monokaryon C6-2-3 (M06-1); Huang1 (D07) and its constituent monokaryon Huang-1-12 (M07-1); Su6 (D08) and its constituent monokaryon Su6-1-2 (M08-1); F2927 (D09); JIN19 (D10); FHJ-12 (D11); JHH (D12) and its constituent monokaryon JIN-1-16 (M12-1); BJP2 (D13) and its constituent monokaryon BJP2-Y-1 (M13-1); F007 (D14) and its constituent monokaryon F007-Y-A (M14-1); F015 (D15) and its constituent monokaryon F015-Y-A (M15-1); WL1073 (D16) and its constituent monokaryon WL1073-Y-3 (M16-1); WS2154 (D17) and its constituent monokaryon WS2154-Y-3 (M17-1); 01922 (D18); JIN4 (D19); FHB01 (D20); FHB07 (D21); FHB021 (D22); F004 and its constituent monokaryon F004-Y-A (M23-1); F006 and its constituent monokaryon F006-Y-A (M24-1) were stored in the Institute of Cash Crops, Hebei Academy of Agriculture and Forestry Sciences. Strain NJ6 and its constituent monokaryon Fv6-3 (As1, as represents the strain was previously released genome assemblies; Liu et al., 2014; Huang et al., 2019); strain 1,123 and its constituent monokaryon W23 (As2) and L11 (As3; Wang et al., 2015); strain C01 and its constituent monokaryon Fv01 (As4) were stored in Fujian Edible Fungi Germplasm Resource Collection Center of China (Yao et al., 2019). The information of strain KACC43778 (constituent monokaryons: KACC42780 and KACC43777) from Konkuk University (Park et al., 2014) and TR19 from Kinki University (Kurata et al., 2016) were referenced. The genome of KACC42780 and TR19 was also used for analysis in this study. The constituent monokaryotic strains of each dikaryon strain in Figure 1 were obtained *via* protoplast-regenerating of dikaryon and isolating monokaryons (Yoo et al., 2004; Huang et al., 2009; Meng et al., 2021). The detailed information including origin, type and color of all the above strains was described in Figure 1, and all the *F. filiformis* strains were maintained at 25°C on potato dextrose agar medium (PDA; 200 g/L potato; 20 g/L glucose; 20 g/L agar).

### Cultivation Conditions

The cultivation of the fruiting bodies of *F. filiformis* dikaryotic strains was performed per the method described by Tao et al. (2019) with some modifications. The cultivation medium was composed of 52.5% cottonseed shells, 15% sawdust, 25% wheat bran, 5% corn powder, 2% calcium sulfate dehydrate, and 0.5% pulverized lime in 60% water. The cultivation bottles containing 200 g sterile medium were inoculated with PDA blocks with mycelia and placed in an incubator at 23°C for mycelia growth (20 days). After the mycelia filled the bottles, the bottles were moved to an incubator at 10°C and 90% humidity to promote primordia formation and fruiting body development.

### Genome Sequencing and Quality Control

Total genomic DNA of *F. filiformis* was extracted by the cetyl trimethyl ammonium bromide (CTAB) method and sequenced using two platforms including Illumina NovaSeq and BGISEQ500 with a paired-end read length of 150 bp. The raw sequencing reads were processed using Fastp (Chen et al., 2018) with default parameters to clean reads with low quality and adapters.

### Reads Mapping and Variants Detection

The clean short reads of all samples were aligned to the reference genome, KACC42780 with 11 complete chromosomes of *F. filiformis* (BioProject ID: PRJNA191921; Park et al., 2014) using BWA-MEM (Li and Durbin, 2010) with the default parameters. The alignment hits of mapped reads were sorted using SAMtools (Li et al., 2009). The statistics and summary of mapping results were performed using the SAMtools stat command. Variant detection and joint genotyping of multiple samples were performed with FreeBayes using the default parameters (Garrison and Marth, 2012).

### Variant Filtering to Obtain Polymorphic Markers

To obtain a set of genetic polymorphic markers, variants were filtered to include: (1) only bi-allelic SNPs, (2) minor allele frequency > 0.05, which removed rare variants, (3) at least two samples with homozygous but different genotypes (i.e., both AA and aa homozygous genotypes were required), (4) a missing rate < 0.2, and (5) no variant sites with heterozygous genotypes in monokaryotic samples. This variant filtering procedure was performed using our custom Perl scripts.

### Variant Calling From Genome Assemblies

The reference genomes of the Fv6-3 (NCBI accession no. PRJNA603211), W23 (NCBI accession no. PRJNA191864), L11 (NCBI accession no. PRJNA191865), Fv01 (NCBI accession no. PRJNA769814), KACC42780 (NCBI accession no. PRJNA191921), and TR19 (NCBI accession no. PRJDB4587) strains were downloaded from the GenBank database of NCBI (Park et al., 2014; Wang et al., 2015; Kurata et al., 2016). Aside from the reference genome KACC42780 used in this study, five other assemblies were chopped into many 5 kb segments with a step of 200 bp when chopping. These chopped segments (simulated long reads) were aligned to the reference genome using Minimap2 (Li, 2018). Variant calls were implemented using BCFtools (Danecek et al., 2021) with the: bcftools mpileup--gvcf, bcftools call, and bcftools merge commands. The allele information for these five assemblies was added to the genotype matrix of the marker set, which contained three types of allele information: missing, 0 (reference allele), or 1 (alternative allele). The haplotype of the reference genome, KACC42780, included all of allele 0 and was also added to the genotype matrix of the marker set.

### Distinguishing Between the Monokaryotic Haplotypes of the Dikaryon

This study included 13 combinations harboring one sequenced dikaryon and one sequenced monokaryon (Figure 1). Genotype phasing of dikaryon and monokaryon haplotype inference in the 13 combinations were performed. The following two procedures were proposed and implemented using our custom Perl scripts: (1) for all SNP sites, the heterozygous genotypes of the dikaryotic strain were phased using the alleles of its sequenced monokaryotic strain, and (2) based on the phased genotypes of the dikaryon, the un-sequenced monokaryotic haplotype was generated as an individual sample.

### Population Structure Analysis

The variant call format (VCF) file of the marker set was converted into PLINK files using PLINK 2.0 (Chang et al., 2015). Based on PLINK files, population structure analysis was performed using ADMIXTURE (Alexander et al., 2009) with a parameter K that ranged from 2 to 6. The PCA analysis was performed using PLINK 1.9 (Purcell et al., 2007) with the parameter—pca 20, and the Identity-By-State (IBS) calculation was also performed using PLINK 1.9 with the parameter—distance square ibs.

## Results

### Whole-Genome Sequencing of the Wild and Cultivated *Flammulina filiformis* Strains

All the 13 *F. filiformis* wild strains (D12-D22, F004, F006) collected from China are yellow strains, and 11 cultivated strains collected from China and Japan contained one brown strain (D01), three white strains (D03–D05), and seven yellow strains (D02, D06–D11; Figure 1). The detailed information including origin, type and color of fruiting body of all the above strains was showed in Figure 1. Of them, dikaryotic strains D01-D22 were performed the whole genome resequencing except F004 and F006 (Resequencing of the genomic DNA of F004 and F006 were failed accidentally), and some available monokaryotic strains of potential value (marked with blue triangle in Figure 1) were also selected to perform the whole genome resequencing. This study describes the whole genome sequencing (WGS) of 22 dikaryotic strains and 17 monokaryotic strains of *F. filiformis* (Figure 1).

A total of 450 Gb sequencing data was generated (Supplementary Table S1), and all strains were sequenced with high depth (above 100-fold, D05 and D13 even reached 1,000-fold). The 39 sets of WGS data involved 22 dikaryotic strains belonging to two categories: wild (*n* = 11) and cultivated (*n* = 11). The dikaryotic strain, Cha01 (D01), had three WGS datasets and the 13 dikaryotic strains (D02–D08 and D12–D17) had two WGS datasets (one dikaryon and one monokaryon, respectively). The remaining eight dikaryotic strains (D09–D11 and D18–D22) and two monokaryotic strains (M23-1 and M24-1) had only one set of WGS data each. WGS datasets of the 39 strains were aligned to the reference genome, KACC42780 (NCBI accession no. PRJNA191921). The overall alignment and coverage rates were 69–83% and 85–93%, respectively (Supplementary Table S2). The presence of unmapped reads may indicate that some genomic components are unique to particular strains, suggesting that one reference genome is not enough to represent the whole population of *F. filiformis*.

### Construction of an SNP Marker Set With High Resolution in *Flammulina filiformis*

**Figure 2:**
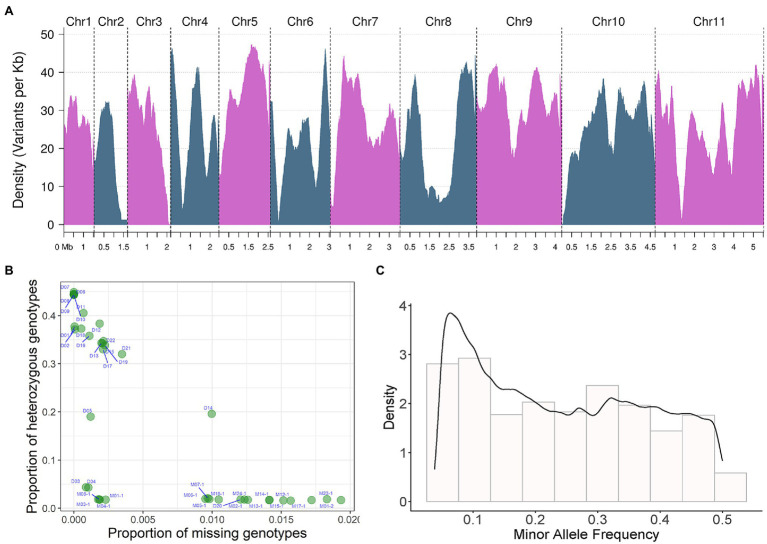
SNP detection and joint genotyping results analysis of 39 sequenced strains. **(A)** Bi-allelic SNP marker density on 11 chromosomes. Density changes of 11 bi-allelic SNP markers on chromosomes. The density of variants per Kb is displayed on the y-axis, the x-axis shows the different chromosomes and their corresponding sequence sizes. Density is the distribution Density of the index recorded on chromosomes, in unit of marker number per KB. Density in **(C)** is the probability Density. The area under the curve is the probability. **(B)** The proportion of missing and heterozygous genotype of each resequenced strain analyzed by quality control (QC) analysis, the proportion of missing genotypes is shown on the x-axis, The proportion of heterozygous genotypes is shown on the y-axis. **(C)** Minor allele frequency (MAF) of bi-allelic SNP.

Through SNP detection and joint genotyping of all 39 sequenced strains (Figures 1, 2), a set of SNP markers with intrapopulation polymorphism, including 849,987 bi-allelic SNPs, was constructed (the strategy for variant filtering is detailed in the Methods, called this marker set as name of SNP850K). Those variants from a strain-specific genomic region not existing in the reference genome were not included in this marker set. However, this marker set SNP850K was basically constructed from shared genomic regions among sampled strains. These bi-allelic SNP markers basically covered all 11 chromosomes (Figure 2A) with a high distribution density of 24.16 SNP markers per kb. Most of the genomic regions (sliding windows of size 500 kb) had more than 10 SNP markers per kb. Sufficient markers with high density and high resolution ensure the validity of subsequent analyses and the representativeness of the study findings.

Using these high-resolution markers, a quality control (QC) analysis of the samples was conducted and the proportion of missing and heterozygous genotypes from each WGS sample was calculated and visualized (Figure 2B). No sample was an extreme outlier. The missing rate was generally low (<2%). The proportion of heterozygous genotypes showed three different levels: high, medium, and low. All monokaryotic strains exhibited a low level of heterozygous proportion (nearly zero), and two dikaryotic strains [D04 (03738) and D03 (Fv093)] also displayed a low level, indicating a high degree of homozygosity between two monokaryons. There were two dikaryotic strains [D05 (03890) and D14 (F007)] with a medium level of ~20%. The rest of the dikaryotic strains had a high level of homozygosity (>30%). We also estimated the minor allele frequency (MAF) of all markers. The histogram showed that there were many variant sites with low allele frequency (Figure 2C).

### Integration of the Reference Genome Assembly of Six *Flammulina filiformis* Strains

The previously released genome assemblies of Fv6-3 (one monokaryon of NJ6 from China), W23 and L11 (two monokaryons of dikaryotic strain 1,123 from China), Fv01 (one monokaryon of C01 which was largely cultivated in Chinese factory and purchased from Japan), KACC42780 (one monokaryon of KACC43778 from Korea), TR19 (dikaryon from Japan) were used for analysis with no extra processing (Figure 1; Supplementary Table S3). Combined with these well-studied strains, an expanded population could help to understand the population structure and breeding history. Each genome assembly was also aligned to the reference genome, KACC42780 (Supplementary Table S3), and the mapped rate was around 85% with a coverage rate between 75 and 84%. After variant detection and genotype identification based on the marker set SNP850K, apart from the reference strain, KACC42780, haplotypes of the other five monokaryotic strains were integrated into the monokaryotic population. The haplotype information (all sites are REF. allele) of the monokaryotic reference genome, KACC42780, was added. A population of monokaryons (*n* = 35) was constructed that contained 17 sequenced and 13 inferred monokaryons, and five monokaryotic reference genome assemblies.

### Population Structure of the *Flammulina filiformis* Dikaryotic Strains

**Figure 3:**
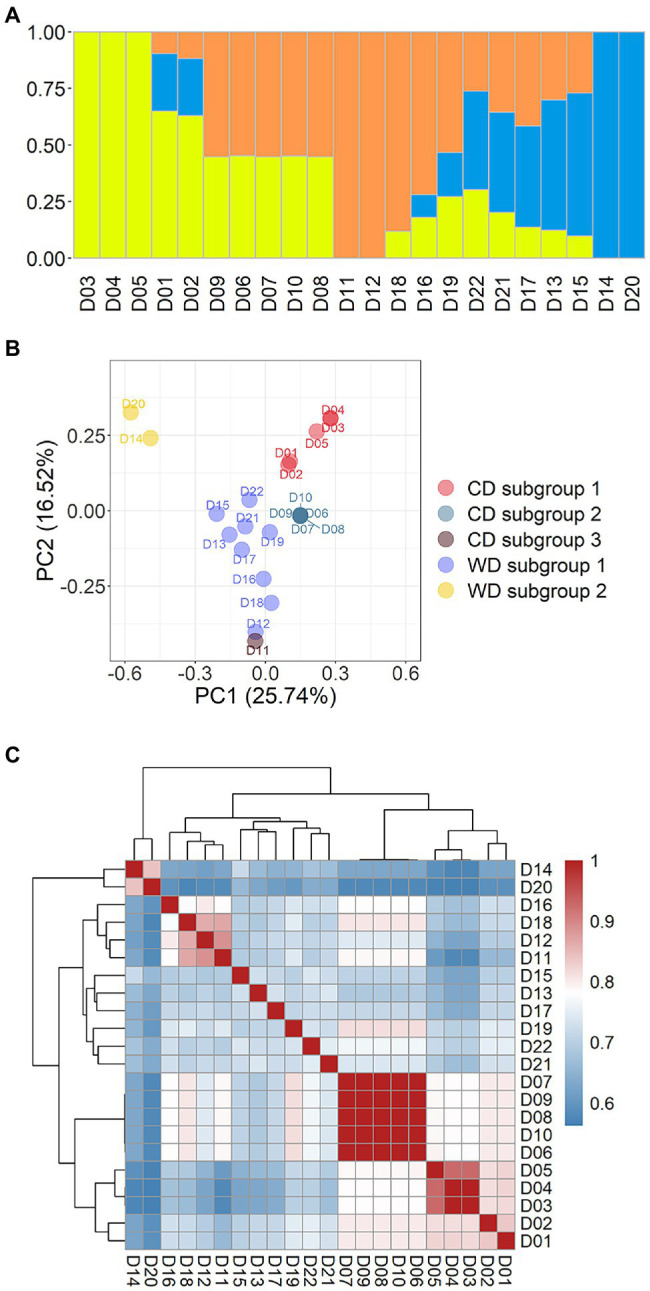
Population structure of 22 *Flammulina filiformis* dikaryons. **(A)** The 22 strains contained 3 ancestral genetic components and their proportions, and different colors represented different ancestral genetic components. **(B)** PCA plot of 22 *F. filiformis* dikaryons. **(C)** Identity-by-Stat (IBS) was used to analyze the genetic relationship of 22 *F. filiformis* dikaryons.

A population containing 22 dikaryotic strains from the 39 WGS datasets was used for admixture and PCA on the marker set SNP850K. Relatedness analysis between strains was also conducted, using the Identity-by-Stat (IBS) method. The result of cross-validation of multiple *K*-values (*K* = 2 to 6) implied that there were most likely three ancestral genetic components in this population (Supplementary Figure S1). Using these results, the 22 dikaryotic strains were divided into three groups (Figure 3). As the respective representatives of the three groups, there were seven non-admixed strains that showed an almost 100% single ancestry component (Figure 3A), while the other strains were admixed. These non-admixed strains were positioned in the outermost area of the PCA plot (Figure 3B), displaying a great genetic distance between different groups. The admixed strains were located in the middle of the PCA plot.

To present breeding history more clearly, the cultivated dikaryotic (CD) strains and the wild dikaryotic (WD) strains were further classified. The CD strains were divided into three subgroups (*n* = 5, 5, and 1, respectively), and the WD strains were divided into two subgroups (*n* = 9 and 2, respectively). The WD subgroup 1 (*n* = 9) contained nine strains (apart from D14 and D20). The WD subgroup 2 (*n* = 2) contained two non-admixed strains (D14 and D20) that were distantly related to the other strains. The population structure and relationship of the wild strains had varied contributions of genetic components that were less related to each other (Figures 3B,C), suggesting that there was high genetic diversity in the wild population.

The three CD subgroups are described below. CD subgroup 1 (*n* = 5) included five strains (D01–D05). There were three non-admixed strains (Figure 3A): D03 (Fv093) and D04 (03878), which had high homozygosity and genetic similarity (Figure 3C), and D05 (03890), which was close but not identical to the other strains. The other two strains D01 (Cha01) and D02 (00117) were also related to them (Figure 3C). The CD subgroup 2 (*n* = 5) included five strains (D06– D10). The PCA result showed that a focused point was overlapped by all five strains (Figure 3B), indicating that they had a highly similar genetic background. High pairwise relatedness scores (IBS values) that were close to 1.0 also confirmed this conclusion (Figure 3C). The two CD subgroups were from the same group and showed strong relatedness (Figure 3C). CD subgroup 3 (*n* = 1) had only one strain, D11 (FHJ-12), which is a newly bred cultivar crossed between a cultivated monokaryon (CM) from the strain D07 (Huang1) and a wild monokaryon (WM) from the wild strain D12 (JHH) in the WD subgroup 1. Thus, D11 (FHJ-12) and D12 (JHH) were related and clustered (Figure 3C) and were close in the PCA plot (Figure 3B). The dikaryotic strain D11 (FHJ-12) was obtained by crossing of monokaryotic strains M07-1 (HUANG-1-12) and M12-1 (JIN-1-16). There was also relatedness between D11 (FHJ-12) and D07 (Huang1).

### Population Structure of *Flammulina filiformis* Monokaryotic Strains

**Figure 4:**
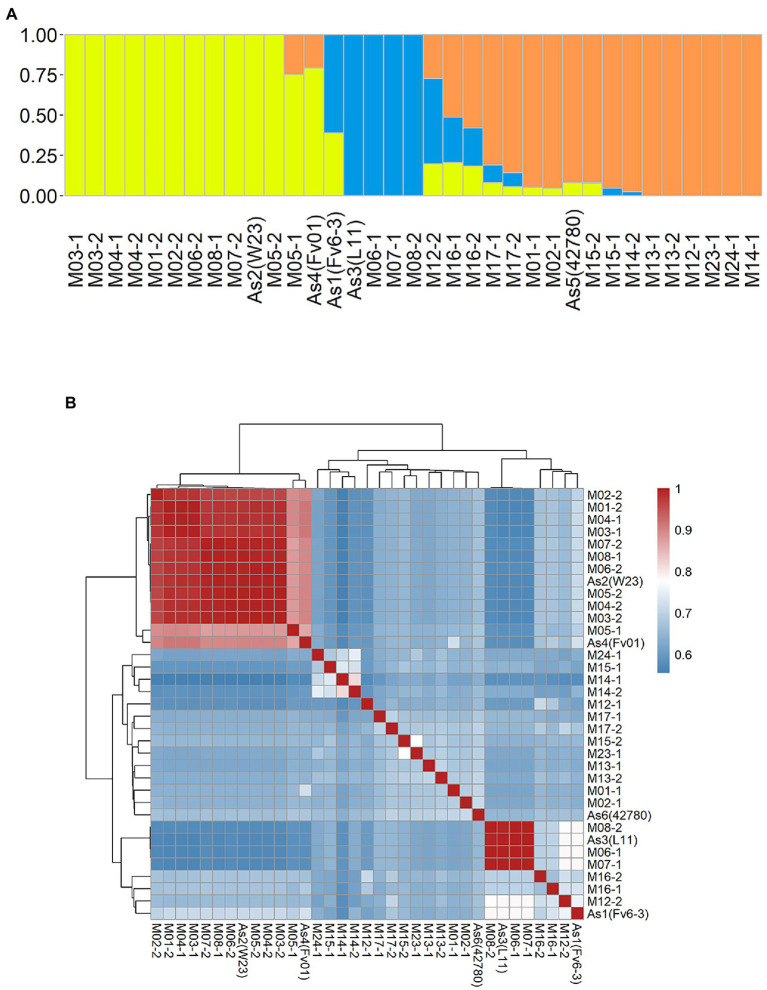
Population structure of 35 *Flammulina filiformis* monokaryons. **(A)** The 35 strains contained 3 ancestral genetic components and their proportions, and different colors represented different ancestral genetic components. **(B)** Identity-by-Stat (IBS) was used to analyze the genetic relationship of 35 *F. filiform is* monokaryons.

An admixture and relatedness for the 35 monokaryotic strains were conducted on the marker set SNP850K. The admixture analysis results clearly showed that there were three well-separated groups (Groups 1–3) that contained 13, 8, and 14 strains, respectively (Supplementary Figure S2; Figure 4A). Each group had a dominant ancestral component and many of the monokaryotic strains were non-admixed (Figure 4A), indicating a 100% contribution from a single ancestral component. There were 12, 4, and 6 (38%) non-admixed strains in the three groups. The cultivated monokaryon (CM) strains primarily came from groups 1 and 2. Moreover, all non-admixed CM strains were highly similar or identical to each other (Figure 4B). The high pairwise relatedness score (IBS values) indicated a narrow genetic background between two monokaryons. Group 3 was almost entirely populated by wild monokaryotic (WM) strains, and the non-admixed WM strains exhibited low pairwise relatedness (Figure 4B). Therefore, it can be concluded that the WM strains were all different from each other, showing a wealth of genetic diversity.

### Comprehensive Analysis of the Population Structure of *Flammulina filiformis* Dikaryotic and Monokaryotic Strains

**Figure 5:**
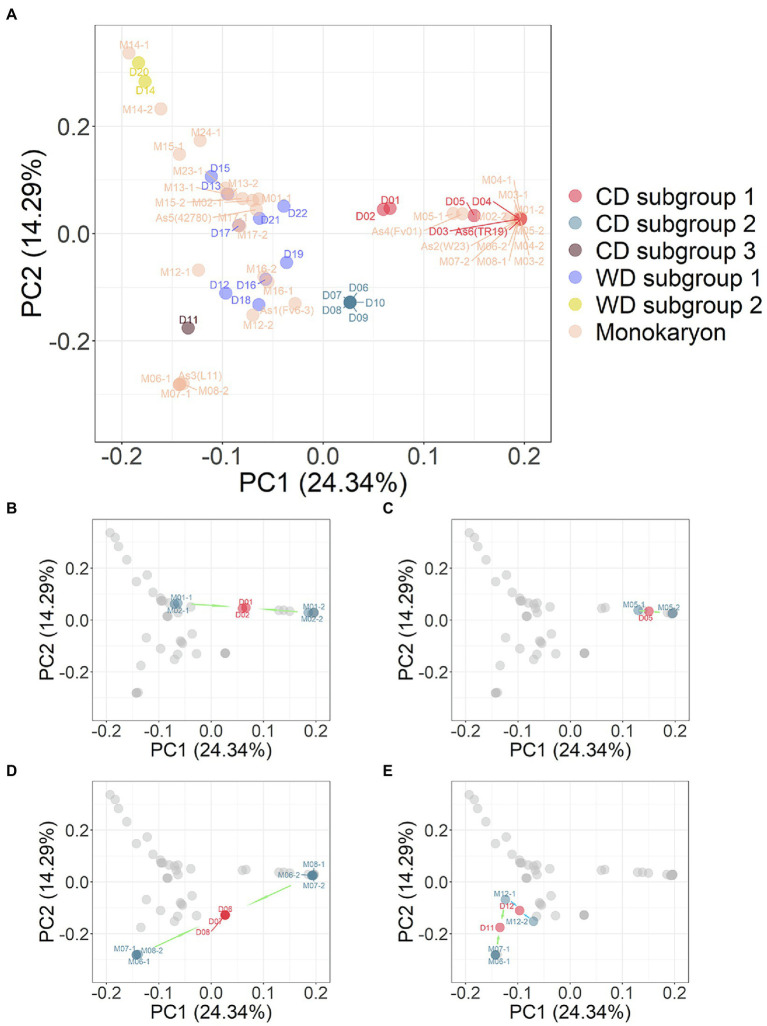
**(A)** PCA plot of combination 23 dikaryotic and 35 monokaryotic strains of *Flammulina filiformis*. The dikaryon strains were denoted by D number, and the monokaryon strains were denoted by M number, and the numbers of dikaryon and the constituent monokaryons strains were consistent. **(B)** Dikaryon strains D01 and D02 and their constituent monokaryon in PCA. The arrow marks represent dikaryon with constituent monokaryons. **(C)** Dikaryon strains D05 with constituent monokaryon in PCA. **(D)** Dikaryon strains D06, D07, and D08 with their constituent monokaryon in PCA. **(E)** Dikaryon strains D11 and D12 with their constituent monokaryon in PCA.

The 35 monokaryotic and 23 dikaryotic strains were combined and PCA analysis was performed on the marker set SNP850K. The results from this integrated PCA analysis (Figure 5) supported the grouping of the strains into three CD and two WD subgroups. The PCA plot (Figure 5A), combined with monokaryons, was consistent with the PCA result of the dikaryotic strains (Figure 3C), e.g., there was the same focused point of CD subgroup 2 [D07 (Huang1) et al]. However, some dikaryons had two monokaryons on either side (Figures 5B–E). In addition, the dikaryon was located at the middle position of its two monokaryons in the PCA plot. Furthermore, for each eigenvector of PC1, PC2, PC3, etc., the value of the dikaryon was equal to the mean of its two monokaryons (Supplementary Table S4). In this study, a total of 14 sets harboring one dikaryon and two monokaryons (Figure 1) had symmetrical monokaryons positions (Figure 5A). This provides evidence for inferring the history of cross-breeding. For example, by using only a dikaryotic population to perform the population structure analysis, it was found that the CD D11 (FHJ-12) strain was closer to the WD D12 (JHH) strain than the CD D07 (Huang1) strain but did not clearly demonstrate the nature of the relationship. In the PCA plot combined with monokaryons, the CD D11 (FHJ-12) strain was in the middle of M12-1 and M07-1, which were the monokaryons of D12 and D07, respectively.

## Discussion

It is important to increase the diversity of *F. filiformis* varieties for the development of the *F. filiformis* industry. First of all, we should clarify the population diversity and structure of existing *F. filiformis* strains. Most previous studies on the population structure of edible fungi, such as *L. edodes*, *F. filiformis*, *A. bisporus* (Lu et al., 2015; Shen et al., 2020; An et al., 2021; Gao et al., 2021; Zhang et al., 2021) have only focused on dikaryons and have not included genetic background analysis of monokaryons in the population. And the origin of domestication and breeding was inferred through the genetic relationship between the wild and cultivated strains in the previous study of population genomics of edible fungi. However, this is not sufficient to make more detailed and specific inferences about the origin of domestication or to reveal the breeding history between closely related strains. Thus, we performed the population structure and PCA analysis of *F. filiformis* dikaryons, monokaryons and combined dikaryons and monokaryons, respectively.

The population structure of the dikaryotic strains just suggests the genetic relationship between the strains, but combining with the haplotype information of monokaryons, it can be used to clearly reveal the pedigree of cultivated strains. In the genetic structure analysis of the monokaryotic population in this study, two clusters were found, and the monokaryons within the cluster were highly related. The PCA result also showed that there were two focused points of these monokaryons, and the focused CM strains were non-admixed. The high similarity of these monokaryons, corresponding to a narrow genetic background, implied that some may be the product of mutant breeding. Accumulating somatic mutations of monokaryons is important for breeding mutants. Based on the symmetrical distribution pattern of the monokaryons, it can be inferred from their location at two focused points that they were crossed to form the CD strains [D07 (Huang1), etc.] that overlapped in a focused point of dikaryons. This inference is consistent with the actual cross-breeding history of *F. filiformis* in China. One of the CD strain “parents” [D07 (Huang1), etc.] was the monokaryon from D03 (Fv093), the backbone material widely used for breeding the white gene. Another “parent,” the monokaryon (L11 etc.) that carried the yellow gene, was also widely used. These two monokaryons are the primary sources currently used for cross-breeding and were probably the first two to be domesticated. Our results provide clues about the domestication origins of these two important monokaryons.

Cross-breeding of the CD strains [D01 (Cha01) and D02 (00117)] was also revealed in the PCA results. The monokaryons of these strains differed, indicating that they produced from different cross-breeding efforts. The CD strains, D03 (Fv093), and D04 (03738) with very short genetic distances were highly similar so that their location on PCA coordinates almost overlapped, suggesting that these strains are highly homozygous strains. The CD strain D05 (03890) was close but not identical to D03 (Fv093) and D04 (03738). By comparing their monokaryons, it was found that the monokaryon (M05-1) was different. The D05 (03890) strain may be the product of back-cross-breeding with the D03 (Fv093) strain as a donor. Comprehensive analysis of the population structure of dikaryons and monokaryons provides important insight into the breeding history, clarifying the pedigree relationships of these cultivated strains and allowing their breeding history to be inferred.

Dikaryons and monokaryons had a distribution regularity in the PCA results; two monokaryons were symmetrically distributed on both sides of corresponding dikaryon. For example, D11 (FHJ-12) was a cross-breeding strain of two monokaryons (M07-1) and (M12-1; Figure 5E), which was consistent with the cross-breeding history of this newly bred cultivar in our laboratory. Thus, we proposed a new research strategy based on analysis of the population structure of the *F. filiformis* genome, which can determine the breeding history and pedigree relationship of cultivated varieties. When we have the genome information of dikaryon and its one of monokaryon, we can infer the information and origin of the other monokaryon. For example, we found that four monokaryons (M06-2, M07-2, M08-1, As2) from D06 (Chuan6), D7 (Huang1), D8 (Su6) and 1,123 were highly similar with the monokaryon M03-2 of white strain D03 (Fv093), and the other corresponding monokaryons (M06-1, M07-1, M08-2) of D06, D7, D8 were highly similar with the monokaryon As3 (L11) isolated from the yellow FL19 strain (Wang et al., 2015). Therefore, the four dikaryotic strains D6, D7, D8, and 1,123 were also highly similar, which were obtained by hybridization of one monokaryon from the white strain and the other monokaryon from the yellow strain. This white strain was originally introduced from Japan, while this yellow strain was originally domesticated from a wild yellow strain collected from southeast (Guo, 1997) China. This deduction was consistent with the actual breeding process of *F. filiformis* in China.

As the genetic lineages of the monokaryon in each dikaryotic strain unclear in *F. filiformis*, the breeding history of each strain cannot be accurately investigated, which can impact the progress of breeding research. Therefore, monokaryon research should be based on of dikaryon research to explore the genetic background and relationship of monokaryons strains. In this study, most of strains were selected to re-sequence the genome of dikaryon and its one constituent monokaryon isolated *via* protoplast manipulation. It can guarantee that the two obtained monokaryons are complete the two parent nuclei for mating. And the monokaryotic haplotypes are more informative and helpful in exploring the origin of a particular strain. We have proposed a strategy to analyze the population structure of monokaryotic strains by studying the genome of *F. filiformis*, in order to effectively guide cross-breeding methods.

**Figure 6:**
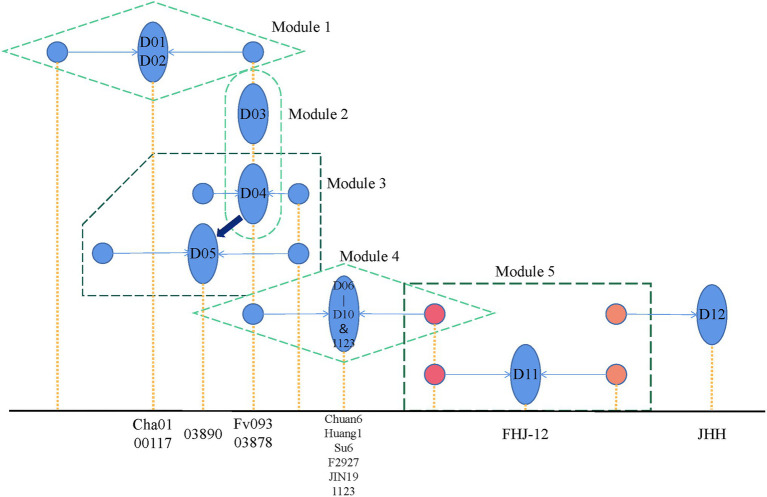
*Flammulina filiformis* breeding history pattern. The blue ellipse represents the dual-nucleated strain, and the two associated monocytes connected by the arrow are represented by the blue circle. The x-axis position corresponding to the yellow dotted line represents the specific strain represented by the figure, respectively.

Here, we summarized the pedigree relationship diagram of *F. filiformis* main strains. As shown in Figure 6, in modules 1 and 4, the strains belonged to CD subgroups 1 2, respectively. The genetic distance between the two monokaryons constituting these dikaryotic strains was greater than other groups, suggesting that the dikaryotic strains were the product of long-term breeding. In module 2, D03 and D04 were shown to be identical by eigenvector of the *F. filiformis* monokaryon and dikaryon. Thus, we believe that the cultivated D03 (Fv093) strain from in Hebei of China, and cultivated D04 (03878) strain from in Japan may be highly similar, which is consistent with the findings that the white strain in China was first introduced from Japan. In module 3, using the eigenvector of *F. filiformis* monokaryon and dikaryon in the PCA plot, D05 (03890) was found to be very similar to D04 (03878) and may serve as its backcross. The fruiting body phenotype of D03 (Fv093) is highly similar with D04 (03878), while D05 (03890) has a longer pileus diameter and a thicker stipe. In module 5, the D11 (FHJ-12) strain was obtained from hybridization of the CD D07 (Huang1) and WD D12 (JHH) strain. The pedigree relationship was consistent with the actual breeding track, and this also verified the reliability of the *F. filiformis* population structure analysis.

According to the pedigree relationship diagram, the hybrids genotypes can be directly phased by the known parental allele, a method called “parental phasing,” which is widely used in plants and animals. Similarly, the dikaryon is a cross between two monokaryons, and its heterozygous genotypes can be phased by the monokaryon allele so that the source of each allele can be distinguished. The phased alleles of variant sites can be used to construct the monokaryotic haplotype. Based on the WGS of the monokaryon, the monokaryotic haplotype can be directly identified. When the dikaryon and the one monokaryon are sequenced, another monokaryotic haplotype without sequencing data can be inferred. When both of the monokaryons are sequenced, the genotypes of their dikaryon can be inferred. In this study, we performed genotype phasing of dikaryon and monokaryon haplotype inference in the 14 combinations that harbored one sequenced dikaryon and one sequenced monokaryon. The two monokaryons (M01-1 and M01-2) of the dikaryotic D01 (Cha01) strain were both sequenced, and the same inference procedures were applied to each. What was inferred agreed with what was actual, indicating the method was effective.

By analyzing the population structure of the *F. filiformis* strains collected in this study, we reached two conclusions. First, among cultivated varieties, we can make inferences about the breeding history of crosses, back-crosses (introgression improvement), and mutant sports varieties because the inferred results remain consistent with available pedigree records (Song et al., 2007; Kang et al., 2011). In future, this strategy can be used to distinguish between two sets of monokaryon haplotypes, providing a strong basis for variety identification, protection, and innovation. This strategy can also be used for other kinds of dikaryotic edible fungi, which is significant for research. In addition, two monokaryons are the main materials for *F. filiformis* cross-breeding, which provides a direction for tracing the origin of *F. filiformis* varieties in China. Second, wild *F. filiformis* strains have rich genetic diversity, which provides valuable genetic resources to help guide future breeding methods (Bao, 2021). Most importantly, an optimal breeding strategy should be designed based on the population structure and pedigree relationship of *F. filiformis* existing strains.

## Data Availability Statement

The authors acknowledge that the data presented in this study must be deposited and made publicly available in an acceptable repository, prior to publication. Frontiers cannot accept a manuscript that does not adhere to our open data policies.

## Author Contributions

HL, WT, BX, and YT conceived and designed the research. HL, LS, YZ, and WX conducted the experiments. HL, LS, WT, XX, WX, BX, and YT analyzed the data and revised the manuscript. HL, LS, WT, and YT wrote the manuscript. All authors contributed to the article and approved the submitted version.

## Funding

This work was supported by grants from the Key Research and Development Planning Project in Science and Technology of Hebei Province (19226368D; 21326315D), the HAAFS Science and Technology Innovation Special Project (2022KJCXZX-JZS-9), the National Natural Science Foundation of China (31902088), the Natural Science Foundation of Fujian Province of China (2019 J01380), the Natural Science Foundation for Distinguished Young Scholar of Fujian Agriculture and Forestry University of China (xjq201919), and the Seed Industry Innovation and Industrialization Project of Fujian Province of China (zycxny2021012).

## Conflict of Interest

The authors declare that the research was conducted in the absence of any commercial or financial relationships that could be construed as a potential conflict of interest.

## Publisher’s Note

All claims expressed in this article are solely those of the authors and do not necessarily represent those of their affiliated organizations, or those of the publisher, the editors and the reviewers. Any product that may be evaluated in this article, or claim that may be made by its manufacturer, is not guaranteed or endorsed by the publisher.
