# Supplementary material for: Comprehensive Genetic Analysis of Monokaryon and Dikaryon Populations Provides Insight Into Cross-Breeding of Flammulina filiformis
Source: Front Microbiol. 2022 Jul 5;13:887259. doi: 10.3389/fmicb.2022.887259 (PMC9294462; doi:10.3389/fmicb.2022.887259)
Supplement: Supplementary file 1 [file Table_1.docx]

**Table S1. Summary of whole genome sequencing (WGS) by NGS**

| Sample name | Type | NGS platform | Data (Gb) |
| --- | --- | --- | --- |
| F007 | Dikaryon | Illumina | 4 |
| F2927 | Dikaryon | Illumina | 4 |
| FHB01 | Dikaryon | Illumina | 4 |
| FHB021 | Dikaryon | Illumina | 4 |
| FHB07 | Dikaryon | Illumina | 4 |
| JHH | Dikaryon | Illumina | 4 |
| JIN19 | Dikaryon | Illumina | 4 |
| JIN4 | Dikaryon | Illumina | 4 |
| SU6 | Dikaryon | Illumina | 4 |
| 03878 | Dikaryon | BGISeq | 15 |
| FHJ-12 | Dikaryon | BGISeq | 15.6 |
| Cha01 | Dikaryon | BGISeq | 18.4 |
| 00117 | Dikaryon | BGISeq | 16.5 |
| 01922 | Dikaryon | BGISeq | 16.5 |
| 03890 | Dikaryon | BGISeq | 26 + 22.5 |
| Chuan6 | Dikaryon | BGISeq | 15 + 5.6 |
| Fv093 | Dikaryon | BGISeq | 16 + 6 |
| Huang1 | Dikaryon | BGISeq | 8.1 |
| F015 | Dikaryon | BGISeq | 15 |
| WL1703 | Dikaryon | BGISeq | 5 + 8.9 |
| WS2154 | Dikaryon | BGISeq | 6 + 20.7 |
| BJP2 | Dikaryon | BGISeq | 26 + 24 |
| F004-Y-A | Monokaryon | BGISeq | 4.04 |
| F006-Y-A | Monokaryon | BGISeq | 4.16 |
| F007-Y-A | Monokaryon | BGISeq | 5.5 |
| F015-Y-A | Monokaryon | BGISeq | 4.5 |
| Fv093-Y-B | Monokaryon | BGISeq | 5.38 |
| SU6-1-2 | Monokaryon | BGISeq | 7.26 |
| JIN-1-16 | Monokaryon | BGISeq | 4.54 |
| C6-2-3 | Monokaryon | BGISeq | 7.3 |
| HUANG-1-12 | Monokaryon | BGISeq | 3.58 |
| CHA-Y-4 | Monokaryon | BGISeq | 7 |
| CHA-Y-25 | Monokaryon | BGISeq | 3.92 |
| WL1073-Y-3 | Monokaryon | BGISeq | 10.76 |
| WS2154-Y-3 | Monokaryon | BGISeq | 10.66 |
| 00117-Y-1 | Monokaryon | BGISeq | 11.36 |
| BJP2-Y-1 | Monokaryon | BGISeq | 12.28 |
| 03878-Y-1 | Monokaryon | BGISeq | 9.4 |
| 03890-Y-1 | Monokaryon | BGISeq | 5.38 |

Note: “+” represent WGS of this sample has different sequencing runs.
