# Supplementary material for: Comprehensive Genetic Analysis of Monokaryon and Dikaryon Populations Provides Insight Into Cross-Breeding of Flammulina filiformis
Source: Front Microbiol. 2022 Jul 5;13:887259. doi: 10.3389/fmicb.2022.887259 (PMC9294462; doi:10.3389/fmicb.2022.887259)
Supplement: Supplementary file 2 [file Table_2.docx]

**Table S2. Summary of short-reads mapping**

| **Sample** | **Total reads** | **Mapped reads** | **Mapped rate (%)** | **Mean of coverage depth** | **Coverage rate (at least 1X)** |
| --- | --- | --- | --- | --- | --- |
| **F007** | **26436510** | **20821296** | **78.76** | **82.62** | **88.62%** |
| **F2927** | **32095670** | **22231454** | **69.27** | **88.69** | **91.29%** |
| **FHB01** | **34426842** | **25570051** | **74.27** | **100.01** | **87.84%** |
| **FHB021** | **29301048** | **22987607** | **78.45** | **91.76** | **90.73%** |
| **FHB07** | **36442672** | **27775060** | **76.22** | **110.08** | **91.79%** |
| **JHH** | **32233924** | **25225592** | **78.26** | **100.75** | **90.65%** |
| **JIN19** | **34270442** | **25337423** | **73.93** | **100.81** | **91.37%** |
| **JIN4** | **31942022** | **24135438** | **75.56** | **95.41** | **91.23%** |
| **SU6** | **34267104** | **25501945** | **74.42** | **101.17** | **91.41%** |
| **00117** | **110461632** | **85313991** | **77.23** | **324.27** | **91.99%** |
| **00117-Y-1** | **75768236** | **60192690** | **79.44** | **228.16** | **88.39%** |
| **03878** | **100619580** | **83121950** | **82.61** | **321.64** | **87.90%** |
| **03878-Y-1** | **62616552** | **51376991** | **82.05** | **201.17** | **87.70%** |
| **03890.r1** | **174979896** | **141327522** | **80.77** | **537.18** | **89.78%** |
| **03890.r2** | **149942870** | **121005918** | **80.70** | **463.74** | **89.69%** |
| **03890-Y-1** | **35888770** | **29293946** | **81.62** | **116.14** | **87.52%** |
| **BJP2.r1** | **174722694** | **137257486** | **78.56** | **515.18** | **91.94%** |
| **BJP2.r2** | **160510164** | **125788021** | **78.37** | **474.15** | **92.17%** |
| **BJP2-Y-1** | **81886932** | **65345070** | **79.80** | **253.26** | **87.68%** |
| **Cha01** | **122671786** | **99741969** | **81.31** | **380.04** | **91.80%** |
| **CHA-Y-25** | **26082752** | **21271386** | **81.55** | **84.97** | **87.18%** |
| **CHA-Y-4** | **46638882** | **37963063** | **81.40** | **149.46** | **87.40%** |
| **Chuan6.r1** | **100590036** | **79642443** | **79.18** | **305.56** | **91.73%** |
| **Chuan6.r2** | **37676632** | **29897983** | **79.35** | **117.75** | **91.43%** |
| **C6-2-3** | **48657124** | **37887807** | **77.87** | **147.70** | **87.81%** |
| **F004-Y-A** | **26930936** | **20991813** | **77.95** | **81.77** | **86.77%** |
| **F006-Y-A** | **27722556** | **21065880** | **75.99** | **82.58** | **87.28%** |
| **F007-Y-A** | **36664806** | **29174757** | **79.57** | **114.20** | **87.12%** |
| **F015** | **100041382** | **78308047** | **78.28** | **290.68** | **92.21%** |
| **F015-Y-A** | **30029674** | **24582229** | **81.86** | **95.07** | **86.98%** |
| **FHJ-12** | **104323224** | **84666537** | **81.16** | **324.32** | **91.06%** |
| **01922** | **110344154** | **83135790** | **75.34** | **317.21** | **92.07%** |
| **Fv093.r1** | **106338064** | **84136133** | **79.12** | **323.38** | **88.30%** |
| **Fv093.r2** | **39212552** | **30964166** | **78.97** | **122.40** | **87.80%** |
| **Fv093-Y-B** | **35839712** | **28495450** | **79.51** | **112.53** | **87.49%** |
| **HUANG-1-12** | **23864076** | **18630494** | **78.07** | **73.54** | **87.69%** |
| **Huang1** | **54570312** | **42903804** | **78.62** | **166.94** | **92.58%** |
| **JIN-1-16** | **30250958** | **24627817** | **81.41** | **98.03** | **86.51%** |
| **SU6-1-2** | **48368670** | **38528350** | **79.66** | **151.42** | **87.56%** |
| **WL1073-Y-3** | **71767198** | **55650943** | **77.54** | **212.62** | **87.30%** |
| **WL1703.r1** | **59535850** | **46932205** | **78.83** | **183.81** | **91.69%** |
| **WL1703.r2** | **33555052** | **26451050** | **78.83** | **104.74** | **91.04%** |
| **WS2154.r1** | **41230596** | **31928268** | **77.44** | **125.22** | **91.64%** |
| **WS2154.r2** | **138390950** | **107379552** | **77.59** | **406.52** | **92.30%** |
| **WS2154-Y-3** | **71120102** | **51006182** | **71.72** | **194.10** | **87.24%** |

Note: suffix of sample name, r1, r2 means different sequencing runs of the same sample.
