# Supplementary material for: Comprehensive Genetic Analysis of Monokaryon and Dikaryon Populations Provides Insight Into Cross-Breeding of Flammulina filiformis
Source: Front Microbiol. 2022 Jul 5;13:887259. doi: 10.3389/fmicb.2022.887259 (PMC9294462; doi:10.3389/fmicb.2022.887259)
Supplement: Supplementary file 3 [file Table_3.docx]

Table S3. Mapping summary of simulated reads from genome assemblies.

| **Sample** | **Mapped rate** | **Coverage rate (at least 1X)** |
| --- | --- | --- |
| Fv01 | 85.51% | 83.52% |
| Fv6-3 | 85.34% | 83.73% |
| L11 | 84.94% | 77.38% |
| W23 | 86.32% | 74.89% |
| TR19 | 86.72% | 81.61% |

Note: these simulated reads generated from the five published genomes were aligned to the reference genome KACC42780, and this table was the summarized result of reads mapping.
