# Supplementary figures and images for: Comprehensive Genetic Analysis of Monokaryon and Dikaryon Populations Provides Insight Into Cross-Breeding of Flammulina filiformis
Source: Front Microbiol. 2022 Jul 5;13:887259. doi: 10.3389/fmicb.2022.887259 (PMC9294462; doi:10.3389/fmicb.2022.887259)

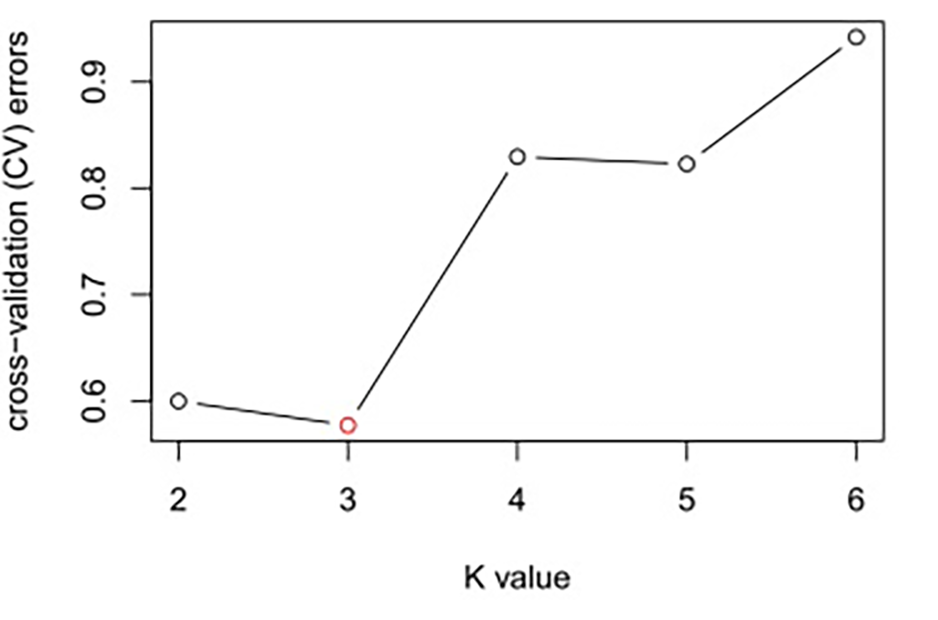

Supplement: Supplementary file 5 [file Image_1.tif]

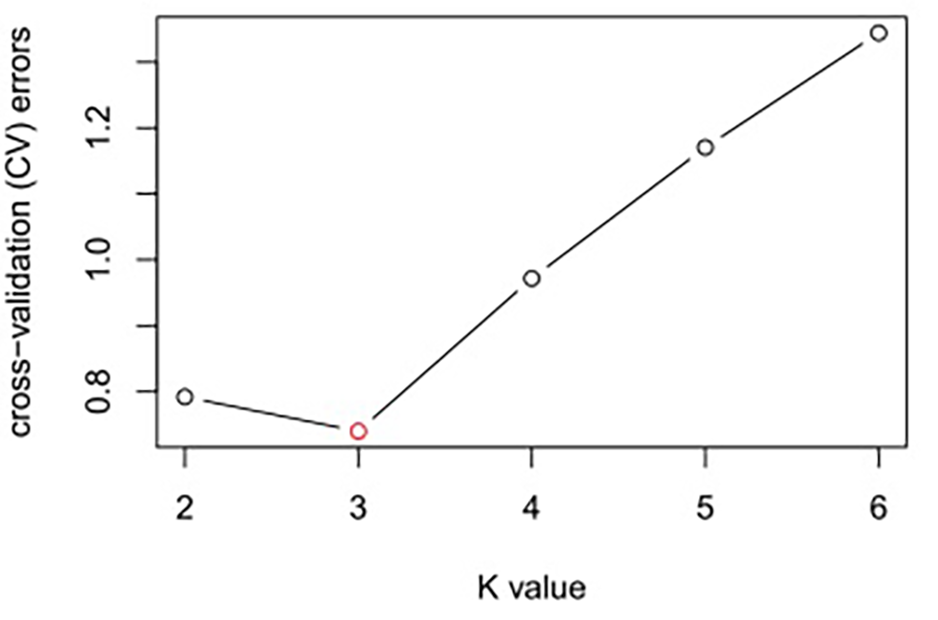

Supplement: Supplementary file 6 [file Image_2.tif]
